# Choroidal metastasis as the presenting feature of a non-small cell lung carcinoma with no apparent primary lesion identified by X-ray: A case report

**DOI:** 10.3892/ol.2014.2389

**Published:** 2014-07-28

**Authors:** MICHAEL LAM, JASON LEE, STEPHEN TEOH, RUPESH AGRAWAL

**Affiliations:** Department of Ophthalmology, National Healthcare Group Eye Institute, Tan Tock Seng Hospital, Singapore 308433, Republic of Singapore

**Keywords:** non-small cell lung carcinoma, choroidal metastasis, chest X-ray

## Abstract

The most common type of intraocular tumor in adults is the metastatic variety, with the choroid as the typical site of involvement. The case of a patient with non-small cell lung cancer, who presented with choroidal metastasis, is described in the current report. In addition, the limitation of using a chest X-ray to identify a large primary lung lesion is highlighted. A 71-year-old female that presented with a choroidal mass lesion is described in the current report. A chest X-ray was conducted and was considered to be normal following detailed investigation, however, a computed tomography (CT) scan of the thorax revealed a large lobulated mass in the right upper lobe. On further histopathological analysis, the patient was diagnosed with a non-small cell lung carcinoma. Thus, when a large choroidal lesion and overlying exudative retinal detachment is observed, a diagnosis of choroidal metastasis should be considered. X-ray images may appear to be normal even in the presence of a large pulmonary lesion. Therefore, in cases where there may be a metastatic lesion, a CT scan is proposed as the optimal diagnostic imaging technique.

## Introduction

The most common type of intraocular tumor in adults is the metastatic variety, with the choroid as the typical site of involvement (1–3). In a survey of patients who presented with an identifiable primary malignancy (3), lung cancer was the most common and breast cancer was the next most common type, as observed in other studies (4–10). In the present report, a case of non-small cell lung cancer presenting with choroidal metastasis is described. Furthermore, the apparently normal chest X-ray images that were obtained highlight a limitation of using X-ray, as a computed tomography (CT) scan subsequently detected the presence of a large primary lung lesion. To the best of our knowledge, there are few cases of choroidal metastasis presenting with non-small lung cancer reported in the existing literature (9).

## Case report

A 71-year-old Malay female was referred to the Department of Ophthalmology, Tan Tock Seng Hospital (Singapore) in October 2011 complaining of blurred vision in the superior field of the right eye, which had persisted for the previous five days. The patient did not exhibit any associated symptoms, such as eye redness, pain or floaters and the patient was not myopic. The patient was admitted to the internal medical unit with suspected pneumonia and was also a carrier of the human immunodeficiency virus for which she was undergoing Highly Active Anti Retroviral Therapy (HAART). The patient had no other medical history. Written informed consent was obtained from the family of the patient.

**Figure 1:**
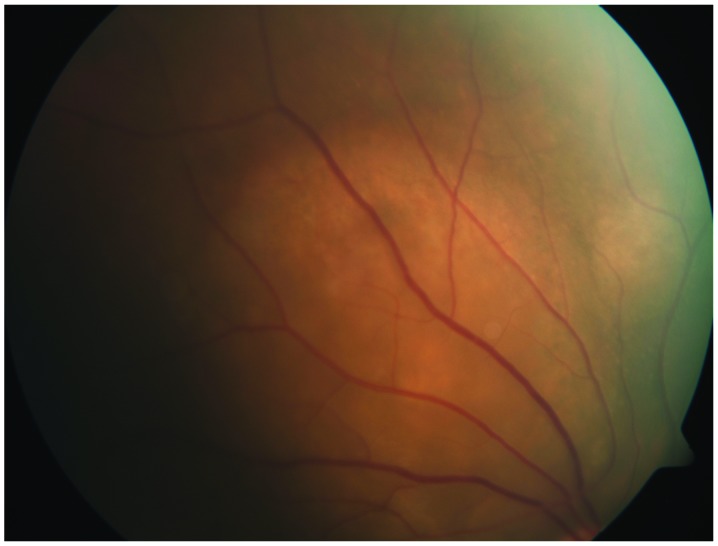
Fundus image demonstrating a large choroidal mass lesion.

Examination revealed best-corrected visual acuity of 6/9 bilaterally. Slit-lamp examination of the anterior segment was unremarkable, however, mild cataracts were apparent. Examination of the posterior segment showed a choroidal lesion spanning ~8 mm and involving the superior half of the macula and the superior temporal quadrant of the right eye, with an overlying area of exudative retinal detachment (Fig. 1). In addition, there was a small choroidal lesion inferior to the lesion. The posterior segment of the left eye was normal. The two optic discs were considered to be normal and had a cup-to-disc ratio of 0.4. The pupillary light reflex was normal, with no relative afferent defects observed and the extraocular movements were normal.

**Figure 2:**
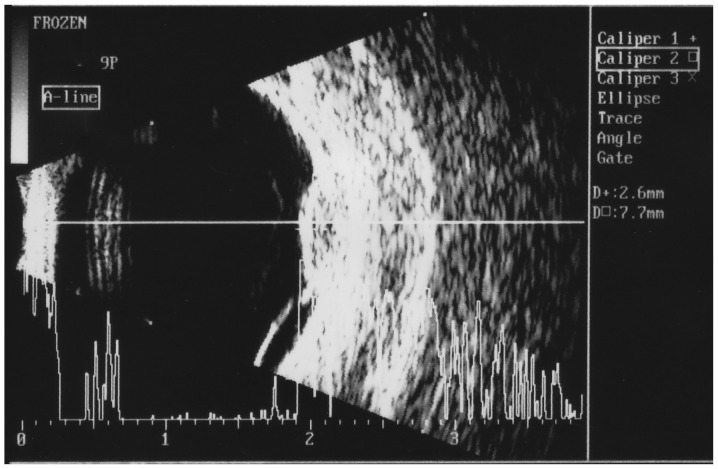
B-scan ultrasound showing a choroidal mass lesion with overlying exudative retinal detachment.

A B-scan ultrasound of the right eye showed the superior temporal lesion measuring 7.7 mm laterally, 9.5 mm radially and a maximum thickness of 2.6 mm, in addition, internal reflectivity was medium to high (Fig. 2). There was an overlying area of exudative retinal detachment. The second choroidal lesion at the inferior equator was small with a maximal thickness of 1.5 mm and medium to high internal reflectivity. Fluorescein angiography demonstrated early blocked hypofluorescence with progressive hyperfluorescence at the border of the lesion in the later phases and pinpointed leakages. Indocyanine green angiography showed blocked hypofluorescence with hyperfluorescence at the border of the lesion.

**Figure 3:**
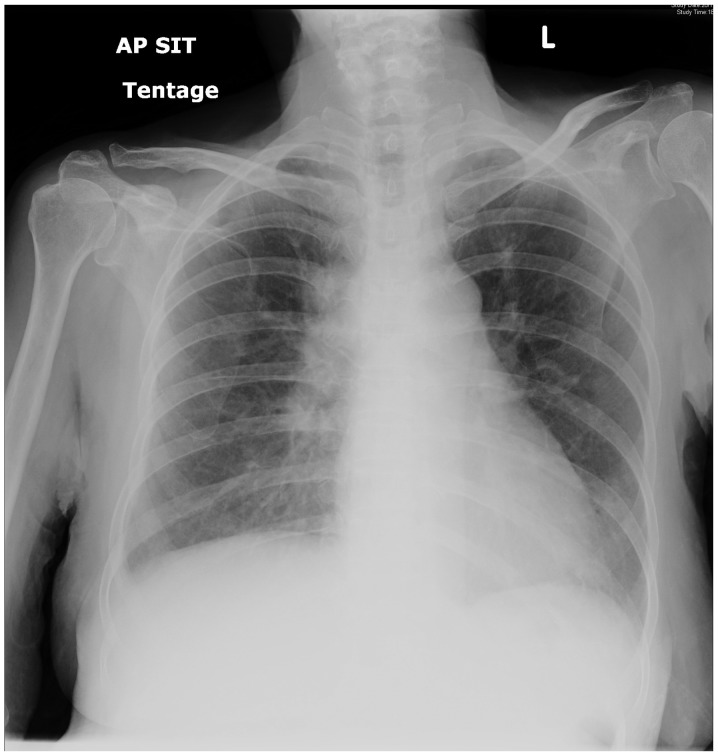
Chest X-ray posterior-anterior view showing no features that indicate a mass lesion.

**Figure 4:**
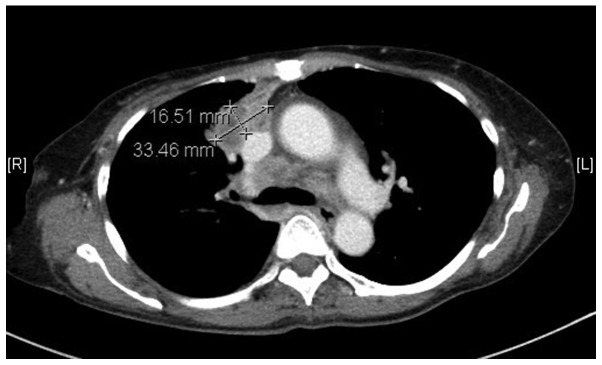
Computed tomography scan of the thorax showing a large lobulated mass in the right upper lobe, measuring 3.3×1.6 cm.

A full blood count indicated normocytic normochromic anemia, however, was otherwise normal. The C-reactive protein level was markedly elevated and the levels of liver transaminases were mildly raised. Serology for syphilis was identified as negative, however, immunoglobulin G serology for the cytomegalovirus and toxoplasmosis were positive. Serum electrolyte levels and the patient’s coagulation profile were normal. Notably, a chest X-ray showed increased air space shadowing in the left lower lobe, which was consistent with pneumonia; the right lung lobes were normal (Fig. 3). It was hypothesized, based on the clinical findings, that the patient was presenting with ocular tuberculosis or choroidal metastasis, therefore, a CT scan of the whole body was performed. The CT scan of the thorax revealed a large lobulated mass in the right upper lobe, measuring 3.3×1.6 cm (Fig. 4). Metastases were indicated by multiple, subcentimeter lesions in the other regions of each lung, as well as by multiple hypodense lesions in the liver and multiple lytic lesions in the thoracolumbar spine. Multiple enlarged bilateral supraclavicular, pretracheal, subcarinal and right hilar lymph nodes were also observed. Thus, the patient was advised to undergo a confirmatory lung biopsy, which revealed a non-small cell lung carcinoma that was indicative of an adenocarcinoma. Immunohistochemistry demonstrated that there were more thyroid transcription factor 1-positive cells than p63-positive cells in addition to faint diffuse staining for the two markers. Immunohistochemistry, therefore, indicated an adenocarcinoma. Although the preservation of the DNA in this case was not sufficient to allow a confident analysis of exon 18, the other three axons of clinical significance were wild-type. Mutations in exons 19 and 21 represented >90% of the total mutations. Furthermore, the *KRAS* mutation was identified in codon 13 of exon 2 and there was no rearrangement of the *ALK* gene. However, pleural fluid cytology did not reveal any malignant cells. Based on the clinical findings, it was hypothesized that the choroidal lesions in the right eye were potentially metastatic. A repeat chest X-ray was conducted two weeks following the initial presentation, which showed interval enlargement of the right-sided pleural effusion and further compressive collapse of the right lung. Bony destruction in the lateral aspects of the right, fifth to seventh ribs and erosion of T11 was noted, which indicated a neoplastic process was occurring. The patient’s family was counseled regarding the terminal nature of the illness and the prognosis of 6–12 months was reiterated to them. Palliative management was advised based on discussion with the family and the patient continued receiving HAART therapy for the retroviral disease. However, the patient succumbed to the cancer within the following two weeks.

## Discussion

Metastatic tumors are the most common type of intraocular malignancy, with the choroid being the typical site of involvement (1–3). The symptoms include blurred vision in 80% of patients, pain in 14%, photopsia in 13%, red eye and floaters in 7% and field defects in 3% (1–4). Metastatic tumors have a creamy yellow appearance and additional clinical features that indicate metastasis are multifocality and bilateralism (4).

The differential diagnosis of choroidal metastasis include choroidal melanoma, choroidal osteoma, choroidal hemangioma, choroidal neovascularization with disciform scarring, tuberculoma and posterior scleritis (2–4).

Common primary sites include the lungs, gastrointestinal tract, pancreas, kidney, the skin, the breasts (in females) and the prostate (in males) (2–4). In a previous study, at the time of ocular diagnosis, 66% of patients reported no history of previous cancer and of the remaining 34%, 49% exhibited a subsequently identifiable primary site (3). In another study, of all of the patients presenting with choroidal metastasis, 58% had lung cancer and 28% had breast cancer (4). However, it was estimated in a study by Kreusel *et al* (5) that in patients with metastatic lung carcinoma, just 7.1% presented with ocular involvement. In the present study, the patient presented with a visual field defect and a fundus examination demonstrated the presence of a large choroidal mass lesion.

B-scan ultrasounds of metastatic lesions regularly show echogenic subretinal masses with diffuse, ill-defined borders and moderate internal reflectivity. Furthermore, overlying retinal detachment is common (4,5). The fluorescein angiographic characteristics of choroidal metastases show early hypofluorescence in the arterial phase, progressive hyperfluorescence in subsequent phases and retinal capillary dilatation at the border of the lesion with persistent pinpoint leakages (4,5). This was consistent with the the observations of the patient in the present study.

The characteristic notable observation in the current case report was an apparently normal chest X-ray in the presence of a significantly large tumor in the right upper lobe. X-ray is routinely conducted by the majority of ophthalmologists to rule out associated pulmonary tuberculosis in patients exhibiting a granulomatous uveitis or choroidal granuloma, however, the use of a normal X-ray (as was used in the current case) may be misleading. Based on this observation, it is proposed that ophthalmologists perform a CT scan to rule out a primary lung lesion rather than advising the performance of a chest X-ray, which may present erroneous findings.

Treatment for ocular metastases is considered to be palliative as the presence of such metastases indicates a hematogenous spread of cancer. Therefore, the aim of any treatment strategy is to maximize the patient’s quality of life and restore or preserve their vision, which may be achieved via radiotherapy or chemotherapy. Surgery is not significant other than for conducting diagnostic biopsies, as often there is no need for tumor debulking and surgery is associated with an increased risk of morbidity.

A review of the literature revealed 12 cases where lung cancer patients were suffering from choroidal metastases as the primary clinical symptom (5–10). In all of these cases, the diagnosis of choroidal metastases indicated the end-stage disease and the cancer dissemination appears to be inevitable; thus, the prognosis is poor and life expectancy is short (5–10). In the present case, on determining the diagnosis and planning the treatment strategy, the patient succumbed due to metastasis. Therefore, the presence of choroidal metastasis in patients with lung carcinoma appears to indicate end-stage disease and is associated with a particularly short life expectancy.

In conclusion, the diagnosis of ocular metastases is based primarily on clinical findings, which may be supplemented by imaging studies and determined via histopathology. Choroidal metastasis should be anticipated when a large choroidal lesion and overlying exudative retinal detachment are observed. An X-ray may appear to be normal even in the presence of a large pulmonary lesion; therefore, in cases of potential metastasis, a CT scan should be performed. The presence of choroidal lesions with the primary lesion in the lungs is indicative of end-stage disease and, therefore, is associated with a limited life expectancy.
